# Pleomorphic adenoma: A diagnostic pitfall in the diagnosis of salivary gland lesions on FNAC: Case reports with review of the literature

**DOI:** 10.4103/1742-6413.70406

**Published:** 2010-09-17

**Authors:** Renuka Gahine, Vijaya Sudarshan, Nighat Hussain, Chandani Krishnani

**Affiliations:** Department of Pathology, Pt. JNM Medical College, Raipur, Chhattisgarh, India

**Keywords:** FNA salivary gland, pitfalls of diagnosis, pleomorphic adenoma, variability of tumor morphology

## Abstract

Fine needle aspiration cytology (FNAC) is commonly being used with increasing frequency for the pre-operative evaluation of salivary gland lesions. However, it has areas of considerable interpretational difficulties. The most frequent problems involve variations in the expected cytology of pleomorphic adenoma (PA). Salivary gland FNACs performed at Pt. JNM Medical College, Raipur, Chhattisgarh, during July 2006 to June 2007 were reviewed, and we report four cases of interesting diagnostic dilemma. As PA is the most common salivary gland neoplasm, it should always be considered and ruled out as the first differential in the diagnosis of salivary gland FNACs. In order to avoid diagnostic pitfalls, we emphasize a diagnostic approach based on the mandatory presence of all three elements of PA, i.e. 3-dimensional cohesive clusters of ductal cells, background of singly lying plasmacytoid myoepithelial cells and dense fibrillary brightly metachromatic stroma with partially obscured entrapped myoepithelial cells. To document the same, we advocate liberal use of repeat aspirations with multiple sampling performed from different parts of the tumor. Some differential diagnostic problems, e.g. carcinoma ex PA, may still however remain insolvable by cytologic means.

## INTRODUCTION

Fine needle aspiration cytology (FNAC) is commonly being used with increasing frequency for the pre-operative diagnostic work-up of salivary gland lesions. However, it has areas of considerable interpretational difficulties. Reasons for these problems include the extraordinary diversity of morphology in salivary gland neoplasms. The most frequent problems involve variations in the expected cytology of pleomorphic adenoma (PA), which is the most common salivary gland neoplasm, representing 45–74% of all salivary gland tumors.[1]

In this retrospective study, records of 50 salivary gland aspirates performed during 1 year, i.e. from 1^st^ July 2006 to 30^th^ June 2007, were reviewed. We report four cases, while dealing with which, we went through agonizing moments of diagnostic dilemma with the potential for clinically important diagnostic errors.

## CASE REPORTS

### Case 1

**Figure 1:**
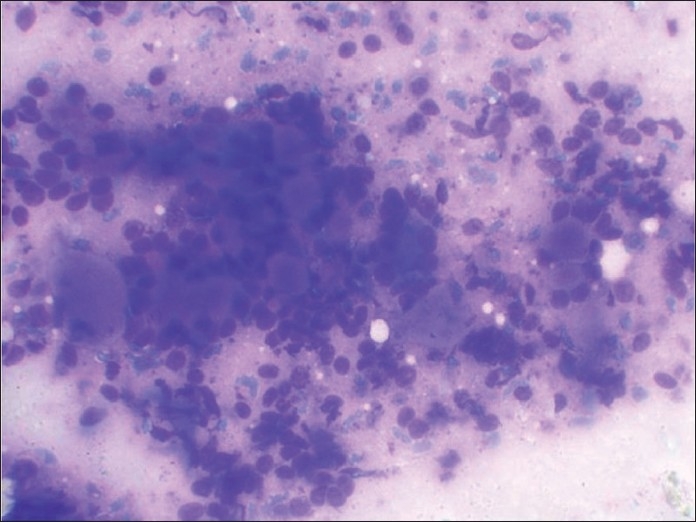
May-Grunwald-Giemsa-stained smear showing predominant pattern of very celebrated appearance of blue cells and metachromatic stroma very typical of cribriform adenoid cystic carcinoma

**Figure 2:**
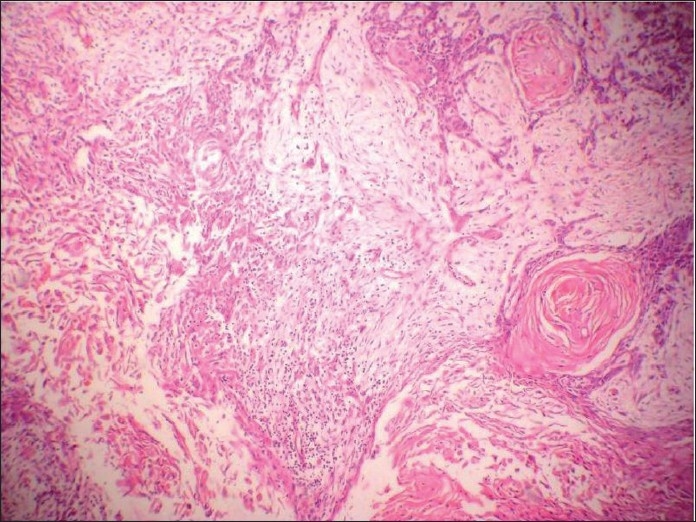
Histologic section of tumor showing squamoid areas at places

**Figure 3:**
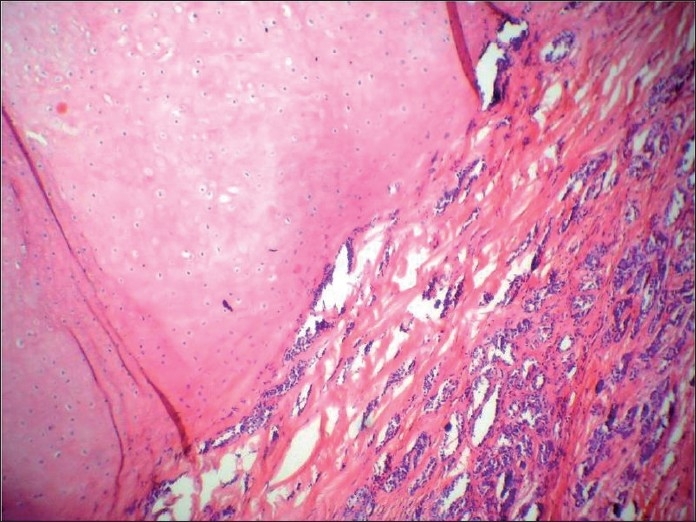
Histologic section showing the very specific feature of pleomorphic adenoma, i.e. close association of epithelial element showing ductal differentiation with cartilaginous tissue, at one place

**Figure 4:**
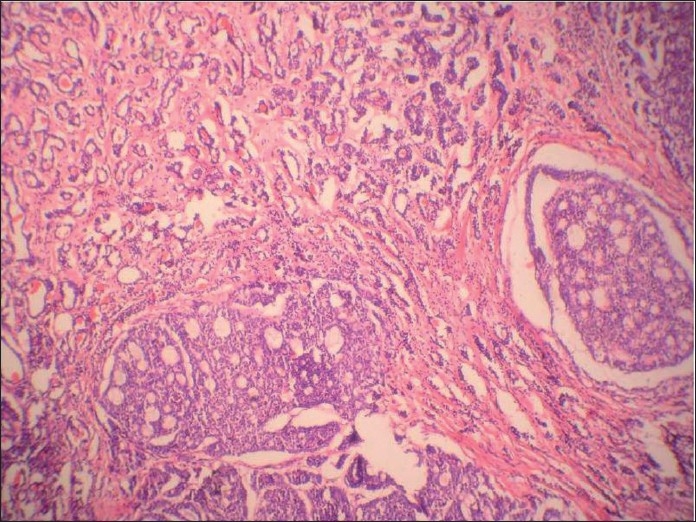
Histologic section showing cribriform arrangement of small tumor cells resembling adenoid cystic carcinoma at another place

**Figure 5:**
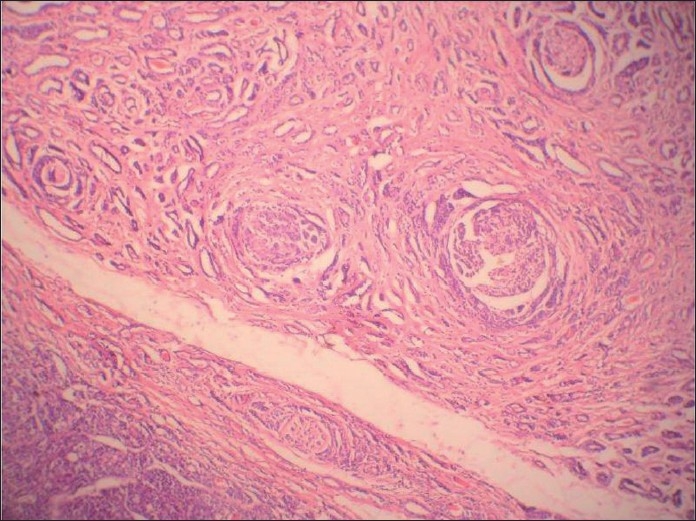
Histologic section showing the very specific feature of adenoid cystic carcinoma, i.e. neural invasion

A 54 -year-old female presented with a slow growing 6 cm X 5 cm painless mass in the left pre-auricular region. The patient had been operated on 10 years back for a swelling at the same site, which was histopathologically proven to be PA. FNA was performed now, which showed cohesive clusters of ductal cells and fragments of myxoid stroma with a fine fibrillar structure. An air-dried preparation showed metachromatic stroma interdigitating intimately with small tumor cells with uniform nuclei with scanty cytoplasm. But, at places, these cells were arranged around small hyaline globules in a pattern suggestive of possible adenoid cystic carcinoma (ACC) [Figure 1]. Repeat aspiration was performed to document the third element of PA. A diligent search revealed plasmacytoid cells. A cytodiagnosis of PA was made based on the above findings. Histologic examination revealed tumor showing epithelial and myoepithelial elements. Squamoid areas were seen at places [Figure 2]. At one place, close association of epithelial element showing ductal differentiation with cartilaginous tissue was also seen [Figure 3]. But, some areas showing cribriform arrangement of small tumor cells was also evident [Figure 4]. A careful search revealed neural invasion in one area [Figure 5]. Hence, a final histodiagnosis of ACC ex PA, was rendered.

### Case 2

**Figure 6:**
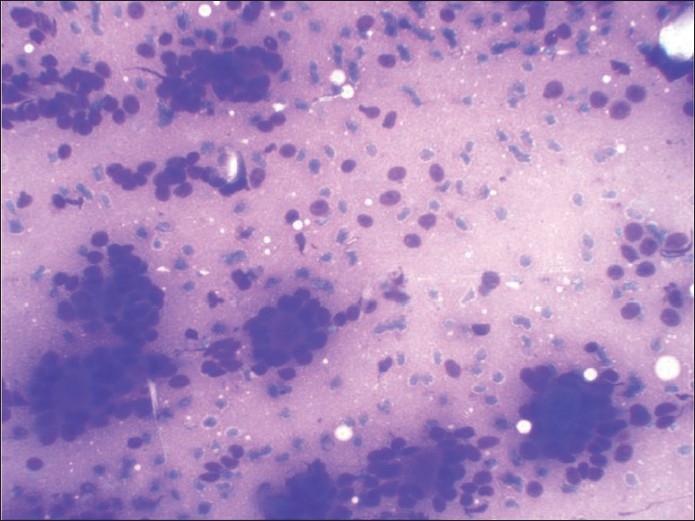
MGG-stained smear showing small blue cells and metachromatic stroma very typical of cribriform adenoid cystic carcinoma

**Figure 7:**
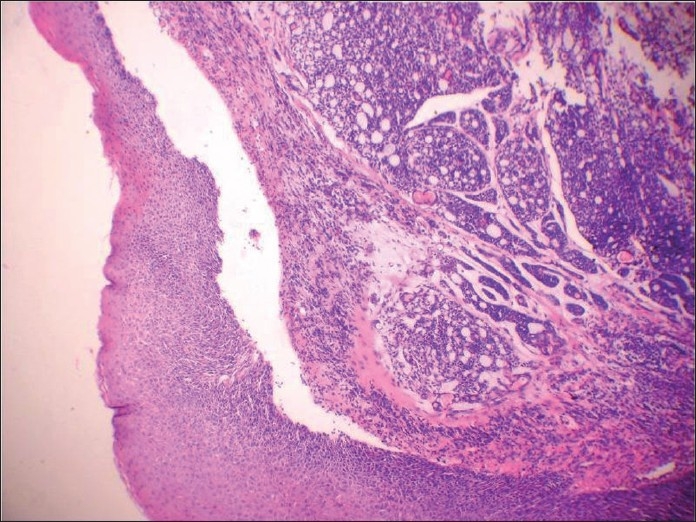
Hematoxylin and eosin-stained section showing encapsulated tumor beneath the normal squamous epithelium, showing extensive adenoid cystic carcinoma-like cribriform areas

**Figure 8:**
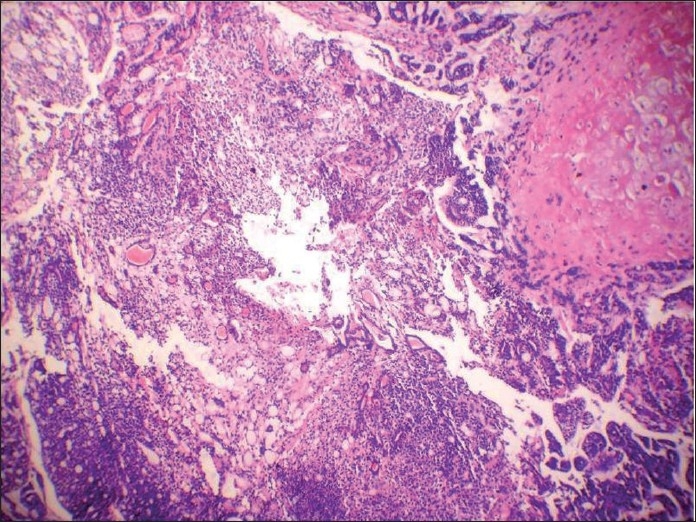
Hematoxylin and eosin-stained section showing cartilagenous association of the epithelial element showing ductal differentiation, confirming the lesion as pleomorphic adenoma

A 72 -year-old male presented with complaints of dysphagia and hoarseness of voice since 3 months. On examination, a growth was seen at the base of the tongue. A computed tomography scan showed a 4 cm × 4 cm mass arising from the posterior third base of the tongue in the midline, which was extending into the surrounding tissues. FNA of the lesion was performed, which revealed a dominant pattern of uniform small blue cells and metachromatic stroma very typical of cribriform ACC [Figure 6]. According to the protocol practiced at our laboratory, repeat aspiration was performed to rule out the remote possibility of PA and more air-dried preparations were made, which showed few chunks of metachromatic stroma interdigitating intimately with small tumor cells. A careful search showed plasmacytoid cells too. Therefore, a cytodiagnosis of PA was made. Pathological examination of biopsy tissue showed an encapsulated tumor beneath the normal squamous epithelium with extensive ACC-like cribriform areas [Figure 7]. But, one of the areas showed a cartilaginous area associated with epithelial elements showing ductal differentiation [Figure 8]. Immunohistochemistry was negative for CD- 117(c-kit). Therefore, a diagnosis of PA with extensive ACC-like cribriform areas was finalized.

### Case 3

**Figure 9:**
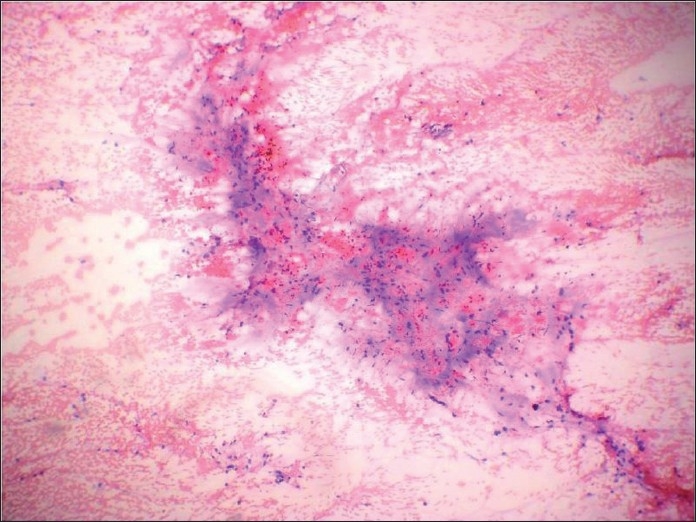
Papanicolaou-stained smear shows a case of malignant peripheral nerve sheath tumor (MPNST) that was incorrectly interpreted as pleomorphic adenoma (PA). This interpretative error was the result of mistaking the eosinophillic stromal matrix of MPNST for the stroma of PA

**Figure 10:**
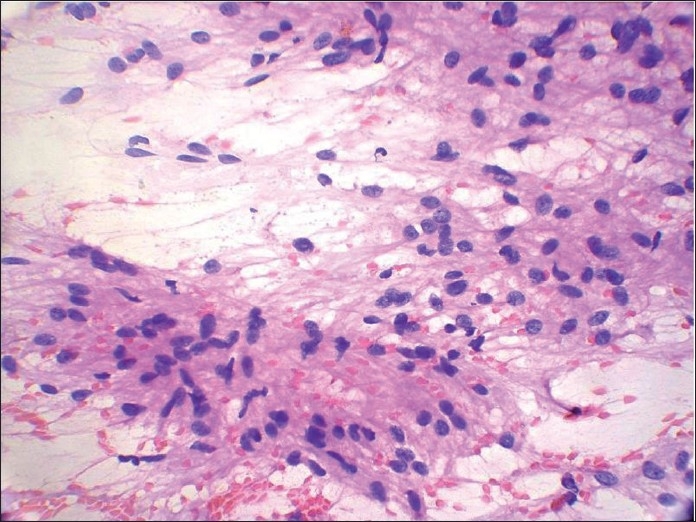
Papanicolaou-stained smear shows spindle cells showing reduced intercellular cohesion with individual cells escaping from the surface of fragments suggesting a malignant lesion. Palisading is seen at places

**Figure 11:**
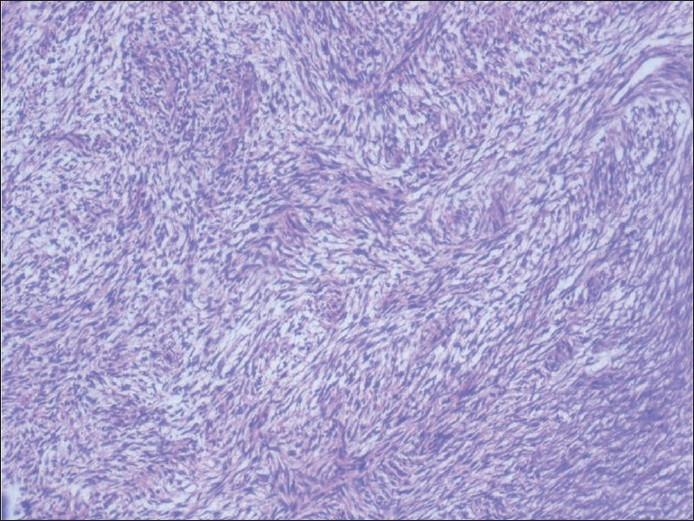
Histologic section of the tumor showing fascicles of malignant spindle cells

A 64 -year-old male presented with a 10 cm × 10 cm painless swelling in the left parotid region since 6 months. It was diagnosed as PA on FNA performed outside. FNA was repeated at our place, which showed cohesive round to spindle cells embedded in an eosinophillic matrix [Figure 9]. Few areas showed reduced intercellular cohesion, with individual cells escaping from the surface of fragments [Figure 10]. Palisading was evident at places. Individual cells showed wavy nuclei with one end blunt and other one tapered with filamentous elongated cytoplasm. Ductal elements could not be demonstrated even on repeated aspirations. On this basis, cytodiagnosis of spindle cell sarcoma, possibly malignant peripheral nerve sheath tumor (MPNST), was considered [Figure 11]. Histopathological examination of the excised tumor revealed features consistent with spindle cell sarcoma.

### Case 4

**Figure 12:**
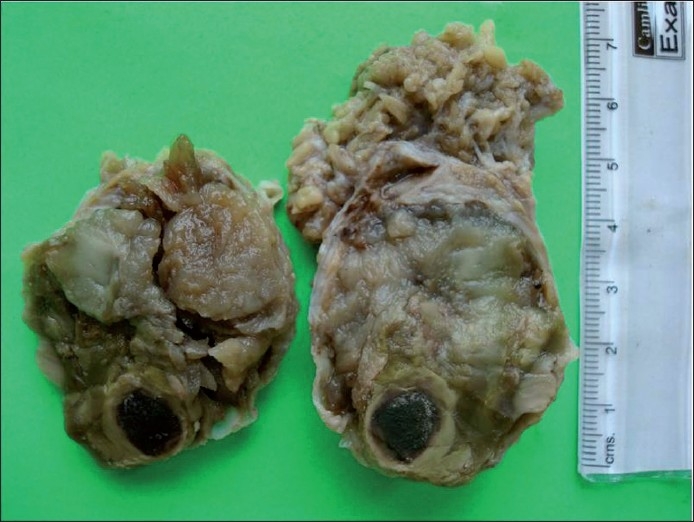
Gross appearance of the tumor showing multiple cysts filled with mucinous material

**Figure 13:**
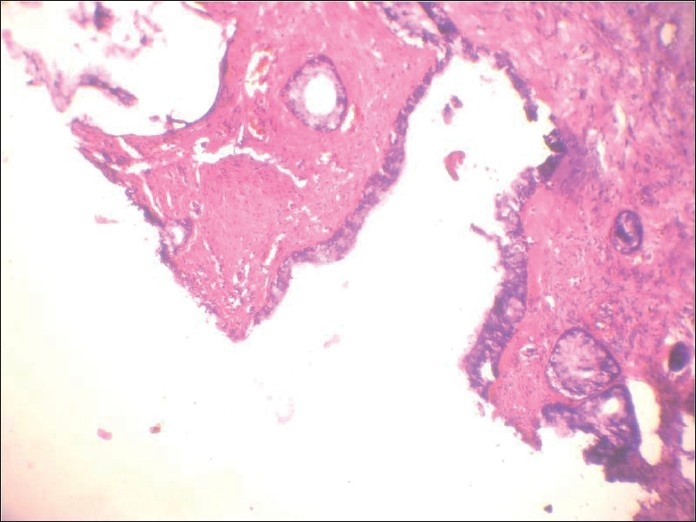
Histologic section of the tumor showing cysic spaces lined by columnar cells showing ciliated metaplasia

**Figure 14:**
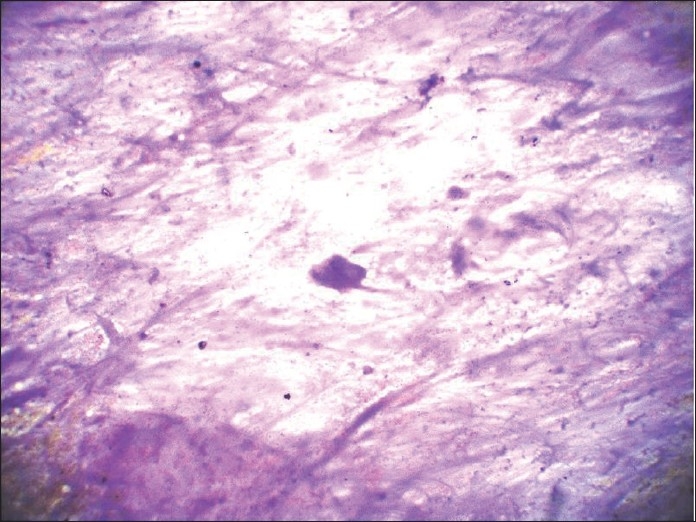
May-Grunwald-Giemsa-stained smear showing mucin, which was misinterpreted as stroma of pleomorphic adenoma, leading to erroneous conclusion

A 56-year-old male presented with painless swelling of the left parotid area measuring 5 cm × 4cm. FNA smears were sparsely cellular, showing a background of fibrillary matrix material. Occasional plasmacytoid cells were seen. Cytodiagnosis of PA was finalized. The excised mass was grossly multicystic [Figure 12]. The histologic examination revealed a cyst wall lined by a single layer of columnar epithelium and fibrous tissue around the mucinous pool with little inflammation. Ciliated metaplasia was evident at places [Figure 13]. Final diagnosis of mucocoele was rendered. Cytology smears were reviewed again. Misinterpreting wispy mucus for chondromyxoid stroma was the reason for misdiagnosis [Figure 14].

## DISCUSSION

**Figure 15:**
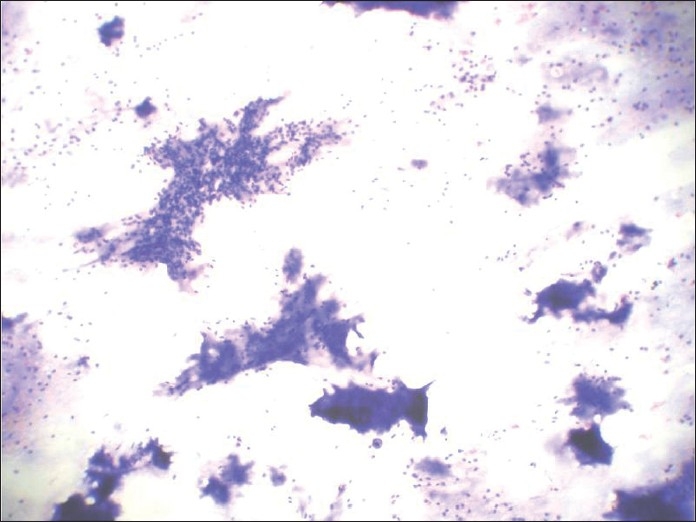
Papanicolaou-stained smear showing dense fibrillary, brightly metachromatic stroma with partially obscured entrapped myoepithelial cells and cohesive 3-dimensional clusters of ductal cells

**Figure 16:**
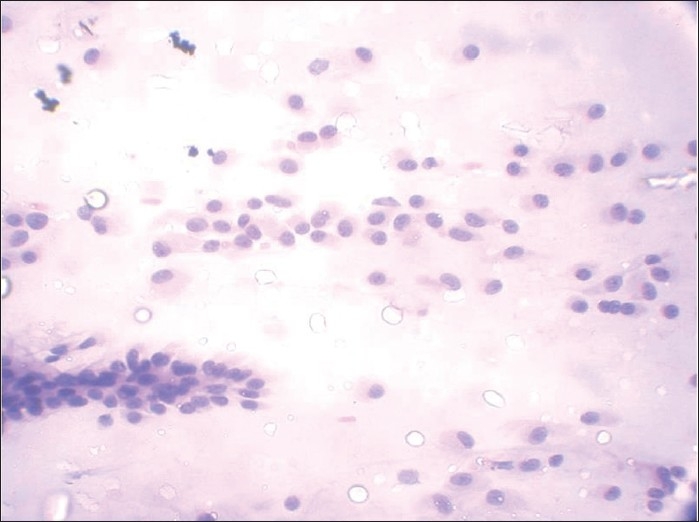
Papanicolaou-stained smear showing dense fibrillary, brightly metachromatic stroma with partially obscured entrapped myoepithelial cells and cohesive 3-dimensional clusters of ductal cells

When pleomorphic adenomas are aspirated, they show various combinations of three elements: ductal cells, chondromyxoid matrix and myoepithelial cells. The latter may be plasmacytoid or spindled [Figures 15 and 16]. Thus, when a salivary gland aspiration shows features of more than one cell component, PA is frequently the correct answer.[2] The chondromyxoid matrix is a more specific and diagnostically helpful feature. In air-dried preparations, myoepithelial cells have pale smudgy blue outlines, being largely obscured by the densely staining matrix. In spite of the difficulty in visualizing these cells on air-dried smears, the appearance of these tissue particles is characteristic and diagnostic.[3]

Although the sensitivity of diagnosing PA by FNA cytology is up to 94%,[4] the differentiation between cellular PA with scant stroma and well-differentiated ACC can occasionally be difficult. PA is perhaps the most common problem in the differential diagnosis of ACC, because both PA and ACC can grow in a cylindromatous or cribriform pattern, complete with hyaline globule formation.[5] The distinction of PA from ACC is especially critical because of significant differences in management. For a benign tumor such as PA, the surgery of choice is superficial parotidectomy with preservation of the facial nerve.[6] For ACC, which is a malignant salivary gland neoplasm with late onset of metastasis and overall poor long-term survival, a total or radical excision of the gland is usually performed, often with sacrifice of the facial nerve.[7]

Careful identification of plasmacytoid myoepithelial cells is the most helpful cytomorphologic feature for distinguishing PA from parotid malignancies, especially ACC.[5] Also, in the benign tumors, the cells interdigitate intricately with the fibrillary connective tissue associated with them. This is in contrast to the sharp interface between tumor cells and extracellular matrix material that forms the spheres and cylinders of ACC.[2] Lowhagen *et al*.[8] advocate that if the cribriform structures appear together with any features of PA, these last findings should be emphasized and a diagnosis of PA should be rendered. They also write that “we refuse to take the full diagnostic responsibility for a radical procedure in which sacrifice of the facial nerve may be necessary in cases where there may be classic cytologic findings of ACC but the patient is symptom free.”

Carcinoma ex PA, was thus underdiagnosed as PA in our case too.

Great care must be exercised in making an unequivocal benign diagnosis based on aspiration of minor salivary glands. To the best of our knowledge, only seven cases of PA involving the base of the tongue have been reported thus far in the literatures. Malignant tumors such as ACC are more common at this site.[9] When the matrix is scanty, the risk of an incorrect diagnosis increases considerably. Identification of small amounts of this material is facilitated by the review of air-dried, Romanowsky-stained slides, where its presence is trumpeted by bright metachromasia rather than the muted translucent quality in fixed preparations.[3] Thus, the cytologic identification of ACC rests on adequate sampling and careful inspection of all material to rule out the possibility of benign PA or basal cell adenoma (BCA).[8] In our case also, we would have misdiagnosed PA at the base of the tongue as ACC had we not gone for repeat aspirations followed by air-dried preparations to highlight the presence of chondromyxoid material.

If the aspirate is evaluated with only a Papanicolaou stain, the myxoid material of pleomorphic adenoma may be mistaken for epithelial mucus. However, mucus lacks the fibrillary quality and staining density of true matrix material.[1] The opposite occurs when the non-specific collagenous stroma of many lesions is thought to represent mixed tumor matrix. Thus, MPNST has been interpreted as pleomorphic adenoma at the time of FNA.[3] Nerve sheath tumors should always be considered in the differential diagnosis of pleomorphic adenoma and a diligent search for epithelial elements is recommended before diagnosing nerve sheath tumors in the head and neck region.[10] Hence, aspirates with little matrix material should be interpreted with caution. Dense fibrillary metachromatically staining matrix material should be sought for.

In summary, FNAC is a fairly accurate pre-operative procedure for the diagnosis of pleomorphic adenomas. Some diagnostic problems do occur in differentiating PA from ACC, BCA, nerve sheath tumors and mucoepidermoid carcinoma. As PA is the most common salivary gland neoplasm, it should always be considered and ruled out as the first differential in the diagnosis of salivary gland FNACs. Complexity and variability of this neoplasm emphasizes the need to base definitive cytologic diagnosis on as many criteria as possible. Therefore, in order to avoid diagnostic pitfalls, we emphasize a diagnostic approach based on the mandatory presence of all three of the following elements of PA before signing out the report: 3 -dimensional cohesive clusters of ductal cells, background of singly lying plasmacytoid myoepithelial cells and dense fibrillary brightly metachromatic stroma with partially obscured entrapped myoepithelial cells. To document the same, we advocate the liberal use of repeat aspirations, with multiple sampling performed from different parts of the tumor. Carcinoma ex PA, is difficult to identify on FNAC. Thus, this differential diagnostic problem may still remain insolvable by cytologic means.

## COMPETING INTEREST STATEMENT BY ALL AUTHORS

No competing interest to declare by any of the authors.

## AUTHORSHIP STATEMENT BY ALL AUTHORS

Each author acknowledges that this final version was read and approved. All authors of this article declare that we qualify for authorship as defined by ICMJE http://www.icmje.org/#author Each author has participated sufficiently in the work and take public responsibility for appropriate portions of the content of this article.

## ETHICS STATEMENT BY ALL AUTHORS

As this is case report without identifiers, our institution does not require approval from Institutional Review Board (IRB) (or its equivalent)

## EDITORIAL / PEER-REVIEW STATEMENT

To ensure integrity and highest quality of CytoJournal publications, the review process of this manuscript was conducted under a double blind model(authors are blinded for reviewers and reviewers are blinded for authors)through automatic online system.
